# Retracing Storage Polysaccharide Evolution in Stramenopila

**DOI:** 10.3389/fpls.2021.629045

**Published:** 2021-03-03

**Authors:** Malika Chabi, Marie Leleu, Léa Fermont, Matthieu Colpaert, Christophe Colleoni, Steven G. Ball, Ugo Cenci

**Affiliations:** ^1^Univ. Lille, CNRS, UMR 8576—UGSF—Unité de Glycobiologie Structurale et Fonctionnelle, Lille, France; ^2^InBioS-PhytoSYSTEMS, Eukaryotic Phylogenomics, University of Liège, Liège, Belgium

**Keywords:** laminarin, glycogen, stramenopila, metabolism, CAZy, polysaccharide

## Abstract

Eukaryotes most often synthesize storage polysaccharides in the cytosol or vacuoles in the form of either alpha (glycogen/starch)- or beta-glucosidic (chrysolaminarins and paramylon) linked glucan polymers. In both cases, the glucose can be packed either in water-soluble (glycogen and chrysolaminarins) or solid crystalline (starch and paramylon) forms with different impacts, respectively, on the osmotic pressure, the glucose accessibility, and the amounts stored. Glycogen or starch accumulation appears universal in all free-living unikonts (metazoa, fungi, amoebozoa, etc.), as well as Archaeplastida and alveolata, while other lineages offer a more complex picture featuring both alpha- and beta-glucan accumulators. We now infer the distribution of these polymers in stramenopiles through the bioinformatic detection of their suspected metabolic pathways. Detailed phylogenetic analysis of key enzymes of these pathways correlated to the phylogeny of Stramenopila enables us to retrace the evolution of storage polysaccharide metabolism in this diverse group of organisms. The possible ancestral nature of glycogen metabolism in eukaryotes and the underlying source of its replacement by beta-glucans are discussed.

## Introduction

Storage polysaccharides are central components of the cell, used to store energy and carbon in an osmotically inert form. They can be found under diverse forms including mannans and glucans; however, by far the two most widespread kinds consist of glucose polymers (glucans) linked by either beta (subsequently called beta-glucan polysaccharides)- or alpha (subsequently called alpha-glucan polysaccharides)-glucosidic bonds. In both cases, storage polysaccharides can be found under either water-soluble polymers (e.g., glycogen and chrysolaminarins) or insoluble solid crystalline material (e.g., starch and paramylon). This difference in physical state of these polymers directly impacts both glucose accessibility (which is slow with solid crystalline granules) and stored amounts (which appear generally higher for crystalline material) as well as the osmotic activity of the cellular compartments concerned by their accumulation (cytosol, vacuoles, and plastid).

All of these storage polysaccharides are obtained through similar kinds of “core” pathways grouped under the term carbohydrate active enzymes (cazymes), which are referenced and classified in families by the CAZy database (Lombard et al., 2014). The glucose is in all cases first activated in the form of a nucleotide sugar (UDP-glucose or ADP-glucose), which is then transferred to a growing chain through glycosyl transferases (GT) behaving as glucan synthases. These consist of GT3 or GT5 enzymes for alpha-glucans (Ball et al., 2011) and GT48 for beta-glucans (Huang et al., 2018). Those synthases will create a link between carbon 1 and 4 and carbon 1 and 3, for alpha- and beta-glucan, respectively. Subsequently, these chains can be branched on position 6 of some glucose residues within the otherwise linear chains. In the case of alpha-glucans, this is usually performed by GH13 subfamilies 8 and 9 (written GH13_8 or GH13_9) glycosyl hydrolases (GH). However, while GH16 activities are thought to be involved in beta-glucan branching (Huang et al., 2016), functional proof of their involvement is still lacking.

Intracellular catabolism of storage polysaccharides is performed either through release of glucose monomers or oligomers (by various hydrolytic activities for both alpha- and beta-glucans), or by phosphorolysis that will produce Glucose-1-phosphate (Glc-1-P), which, unlike glucose monomers, retains one of the two high-energy bonds used during glucan synthesis. In alpha-glucan metabolism, this is performed by GT35 (Wilson et al., 2010) phosphorylases, while for beta-glucan degradation, GH94, GH149, and GH161 have all been reported to perform phosphorolysis (Kitaoka et al., 2012; Kuhaudomlarp et al., 2018, 2019) *in vitro.* Again, their *in vivo* functional involvement still requires demonstration. To complete degradation, enzymes performing the reverse reaction of branching called debranching enzymes are needed. For alpha-glucan degradation, two distinct types of enzymes are reported. These include both so-called “direct” and indirect debranching enzyme. Direct debranching enzymes of GH13 subfamily 11 are widespread enzymes of bacterial glycogen degradation while their distribution remains restricted to Archaeplastida within the eukaryotic domain where they play a role in both synthesis and degradation of starch (Mouille et al., 1996; Cenci et al., 2014). Such enzymes “directly” access the α-1,6 branch and hydrolyze it, thereby releasing oligosaccharides in the cytosol. Indirect debranching enzyme (iDBE) defines a bifunctional enzyme selectively active for eukaryotic glycogen degradation. It includes two domains, a GH13_25 associated with a GH133 domain (Teste et al., 2000). Catalysis proceeds first by transfer of chain, segments preceding the branch, followed by the hydrolytic cleavage of the unique glucose residue left at the branch thereby obviating oligosaccharide release. However, no activity has been proposed, yet, for debranching in the case of beta-glucans.

While all steps of the core pathway of alpha-glucan metabolism have received abundant functional demonstration of their involvement in many distinct lineages, functional evidence supporting the core pathway of beta-glucan metabolism remains scarce and mostly limited to the glucan elongation step (Huang et al., 2016, 2018). Caution is thus needed as many of the core pathway inferred steps still require functional demonstrations.

The much wider distribution of the alpha-glucan metabolism pathways in the eukaryotic tree of life [all unikonts (Amoebozoa, Obazoa) and many bikonts (SAR, Cryptista, Haptista, Archaeplastida, Excavata)] when compared to that of beta-glucans (a few bikonts) may suggest that alpha-glucan metabolism defines a comparatively more ancient eukaryotic pathway.

In addition, it is worth noting that alpha- and beta-glucans are also found in cell walls. While cell walls with beta-1,3 glucan polysaccharides are quite common, cell wall containing alpha-1,4 glucans are quite rare being presently restricted to mycobacterium (Dinadayala et al., 2008) and a very small number of fungi (Yoshimi et al., 2017).

However, the more the diversity is explored, the more complex the picture gets. Among others, Stramenopila have been known chiefly as a beta-glucan storage polysaccharide accumulating organism, but recent analysis has shown additional layers of complexity in some lineages (presence of alpha-glucan storage metabolic enzymes in Blastocystis) (Gentekaki et al., 2017).

**FIGURE 1:**
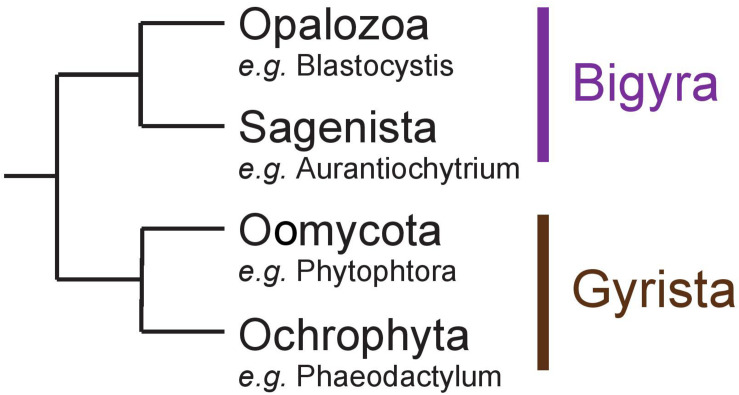
Schematic representation of the Stramenopila evolution tree.

Stramenopila are a very diverse group of eukaryotes including both photosynthetic and free-living phagotrophic or non-phagotrophic or parasitic heterotrophic lineages. In all cases, unicellular species such as diatoms or intestinal Blastocystis protists as well as more or less complex multicellular clades such as brown algae and oomycete pathogens have been reported. Several hypotheses have been proposed to explain the evolution of Stramenopila, including a unique secondary red alga endosymbiosis (Chromalveolate hypothesis) (Cavalier-Smith, 1999) or a succession of endosymbioses where Haptophyta would have been internalized by a Stramenopila non-photosynthetic protist ancestor (Serial hypothesis) (Baurain et al., 2010). Non-photosynthetic Stramenopila with no evidence for a prior endosymbiotic history have been identified including intestinal protists (Blastocystis) or multicellular parasites (e.g., Oomycetes) (Stiller et al., 2014). Stramenopila can be separated into two clades: (i) Bigyra subsequently composed of Opalozoa (e.g., Blastocystis and Halocafeteria) and Sagenista (Aurantiochytrium and Aplanochytrium), and (ii) Gyrista composed of Oomycota (e.g., Phytophtora) and Ochrophyta (e.g., the multicellular brown alga Ectocarpus and the unicellular diatom Phaeodactylum) (Derelle et al., 2016; Figure 1). The Ochrophyta are presently believed to account for up to 25% of Earth’s total annual carbon fixation chiefly through the metabolic activity of diatoms (Field, 1998). In diatoms, this carbon fixation is known to occur through storage in vacuoles of glucose polymers in the form of chrysolaminarin polysaccharides consisting of beta-1,3-glucans with occasional beta-1,6-linked branches. In other Stramenopila from the Gyrista group, storage polysaccharides of very similar structures have been named mycolaminarin and laminarin in Oomycota (Kamoun, 2003) and brown algae (Graiff et al., 2016), respectively. However, in Opalozoa, despite the fact that very little is known on the nature of their carbon storage, glycogen accumulation has been reported in Blastocystis intestinal protists. As to Sagenista, current literature survey points to a major accumulation of storage lipids, as some species are described for their high quantity of polyunsaturated fatty acid (Lee Chang et al., 2012). However, storage lipid accumulation is often found in Stramenopila, including groups that are known to accumulate storage polysaccharides. No clear cytological or genomic analysis has really, to our knowledge, questioned the possible presence of storage polysaccharide metabolism in Sagenista. In addition, in Opalozoa, despite the recent preliminary report of alpha-glucan metabolism in Blastocystis (Gentekaki et al., 2017), the paucity of studies dealing with such issues prevent us from fully understanding carbon storage in these organisms.

We now infer the distribution of these polymers in Stramenopila through the bioinformatic detection of their suspected metabolic pathways and confirm the presence of alpha-glucan metabolism in Opalozoa other than Blastocystis, as well as the apparent absence of both alpha and beta storage polysaccharide metabolism in all sequenced Sagenista genomes. We further analyze the origin of beta-glucan metabolism in Gyrista and propose that the Stramenopiles ancestor would have been able to synthesize both alpha- and beta-glucan storage polysaccharides.

## Materials and Methods

### Sequence Selection

Sequences were selected from a selection of Stramenopiles selected for their completeness and their low contamination used from Vlierberghe et al. (2021), as well as from other sources to ensure a representative diversity of Stramenopiles (Corteggiani Carpinelli et al., 2014; Harding et al., 2016). In addition, we used the advance access genome drafts for Ochromonas (https://genome.jgi.doe.gov/Ochro2298_1/Ochro2298_1). Genomes selected are as follows: *Albugo candida, Aplanochytrium kerguelense, Aurantiochytrium limacinum, Aureococcus anophagefferens, Blastocystis* sp*., Ectocarpus siliculosus Fragilariopsis cylindrus, Nannochloropsis gaditana, Ochromonadaceae* sp. CCMP2298, *Halocafeteria seosinensis, Phaeodactylum tricornutum Phytophthora infestans, Pseudo-nitzschia multiseries, Pythium ultimum*, *Saprolegnia parasitica, Schizochytrium aggregatum*, and *Thalassiosira pseudonana*. Every proteome was then screened using dbCAN hmm model (Yin et al., 2012), which is based on the CAZy database (Lombard et al., 2014) using HMMER (Mistry et al., 2013), for specific models suspected of being involved in either alpha storage polysaccharide and beta storage polysaccharide. In addition, we have selected sequences from the CAZy database to design our own profiles for each GH16 subfamily using MUSCLE software (Edgar, 2004) to align sequences and hmmbuild to construct profiles (Mistry et al., 2013). We then used a threshold of 10e-40 to annotate the sequence automatically; in addition, we consider classification with subfamilies only if the second best-matching HMM had an E-value more than 10e-10-fold greater. Then, we manually look for annotation between 1e-40 and 10e-20, as well as check subfamilies with less than 10e-10-fold greater.

### Deciphering New Putative Enzymes Involved in (Chryso-myco)laminarin

To decipher new putative enzymes involved in (chryso-myco)laminarin biosynthesis in Stramenopiles, we used the literature on beta-glucan metabolism to select a wide range of CAZy proteins putatively involved in the beta storage polysaccharide metabolism in Stramenopiles. Using the annotation process described above, we then compared their distribution to GT48 and GH16_2 (the only two enzymes known to be involved in laminarin metabolism). If the distribution was correlating with GT48 and GH16_2, we considered it as new putative enzymes. As criteria for correlation, we considered a CAZy family (or subfamily) if (i) no organisms without GT48 and GH16_2 would have this group of enzymes, and (ii) at most two genomes, from the known beta-glucan storage polysaccharide accumulator, lack a family.

### Subcellular Localization Prediction

In order to predict the subcellular locations of beta-glucan metabolism enzymes, we first selected sequences from our annotation survey (Supplementary Table S1) for GT48, GH16_2 and GH5_33, GH81, and GH161. Then, we carried out different predictions using a combination of Almagro Armenteros et al., 2019; Petsalaki et al., 2006, and TMHMMv2.0 (Krogh et al., 2001) under the “non-plant” modes.

### Phylogenetic Analysis

For the CAZy families considered as key enzymes for storage polysaccharide metabolism (alpha-glucan storage polysaccharide metabolism enzymes: GH13_25-GH133, GT35, GT5, and GH13_8; beta-glucan storage polysaccharide metabolism enzymes characterized: GH16_2, GT48, and the putative enzymes associated with beta-glucan storage polysaccharide GH5_33, GH81, and GH161), we have performed phylogeny using a pipeline used in previous studies (Cenci et al., 2016, 2018a). We used all sequences, annotated as mentioned above, and formed a cluster for each different CAZy family. Each cluster was used to retrieve sequences using homology searches by BLAST against sequences of the non-redundant protein sequence database of the NCBI and sequences from other databases (MMETSP and data publicly available). We retrieve the top 2,000 homologs with an E-value cutoff lower than 1e-10 and aligned them using MAFFT with the quick alignments settings (Katoh and Standley, 2013). Block selection was then performed using BMGE (Criscuolo and Gribaldo, 2010) with a block size of 4 and the BLOSUM30 similarity matrix. We generated preliminary trees using Fasttree (Price et al., 2010), and “dereplication” was applied to robustly supported monophyletic clades using TreeTrimmer (Maruyama et al., 2013) in order to reduce sequence redundancy. The final set of sequences was manually selected and focused around Stramenopila sequences. Finally, proteins were re-aligned with MUSCLE, block selection was carried out using BMGE with the same settings as above, and trees were generated with Phylobayes-4.1 under the catfix C20 + Poisson model with the two chains stopped when convergence was reached (maxdiff <0.1) after at least 500 cycles, discarding 500 burn-in trees. Bootstrap support values were estimated from 100 replicates using IQ-TREE under the LG4X model and mapped onto the Bayesian tree.

## Results

### Detection of Alpha-Glucan Metabolism in Opalozoa

In a previous study of the Blastocystis cazyme content, we have uncovered several genes known in other eukaryotes to be involved in alpha-glucan storage polysaccharide metabolism (Gentekaki et al., 2017). However, the scarcity of genomes in Opalozoa and the evolutionary trajectory of Blastocystis compared to other Stramenopila [a history marked by high-speed diversification and LGT (Eme et al., 2017)] prevented us from concluding the ancient nature of alpha-glucan metabolism in Stramenopila. Thus, we added a recent transcriptome of another Opalozoa: *H. seosinensis*, to the analysis and found the presence of all required alpha-glucan metabolism enzymes in these organisms (Table 1). We, thus, found a full set of enzymes able to perform glycogen biosynthesis including a GT5 glycogen synthase, a branching enzyme (GH13 subfamily 8 written: GH13_8), and an indirect debranching enzymes with the two expected domains (GH13_25-GH133) as well as glycogen phosphorylase (GT35), all of which are present in both Blastocystis and Halocafeteria. However, those enzymes were never found in other Stramenopila from either the Gyrista or the Sagenista sister group, suggesting the absence of alpha-glucan storage polysaccharide metabolism in these taxa.

**TABLE 1:**
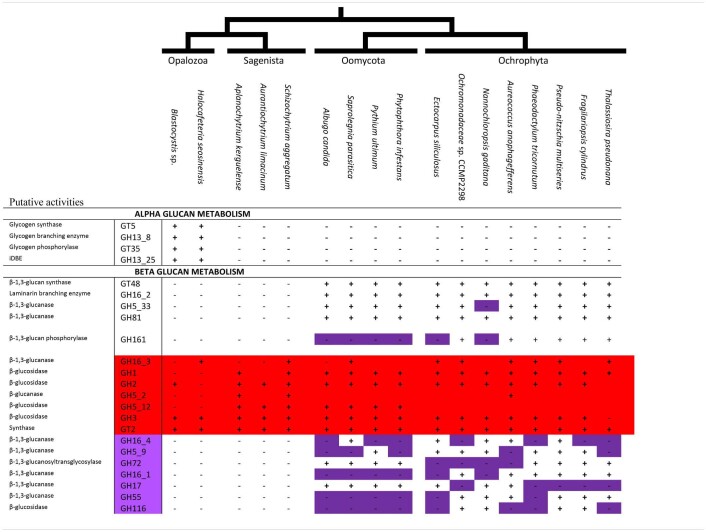
Table of CAZy families (indicated on the first column) and subfamilies found in 17 Stramenopila genomes.

*Presence of those enzymes in the genome is indicated by a +, while absence is indicated by a - and a purple color. To better observe correlation for beta-glucan storage metabolism, we also added a red background to highlight enzymes not specifically linked to beta-glucan metabolism, because they are present in organisms without beta-glucan storage polysaccharides, even if some isoforms can probably act on it. In addition, we display in light purple activities that were missing in clade normally synthesizing beta-glucan storage polysaccharides. For CAZy families and subfamilies, we indicate with a white background if they were linked to one of the metabolisms. Despite a low correlation for GH161, we kept it because of its putative involvement as stated in Kuhaudomlarp et al. (2019). Finally, we have indicated on the first column putative activities of each cazyme.*

### Identification of Strong Candidate Enzymes Involved in (Chryso-myco)laminarin Biosynthesis in Stramenopila

Alpha-glucan metabolism being limited to Opalozoa, we further checked for the presence of candidate enzymes possibly involved in beta-glucan metabolism in Stramenopila. The GT48 characterized in *P. tricornutum* (Huang et al., 2018) has been functionally demonstrated to be active for synthesis of linear beta-1,4 glucans while a GH16 is presently highly suspected but not fully demonstrated to be active for synthesis of the beta-1,6 branches. We can thus correlate the presence of beta-glucan metabolism to the presence of the GT48. In addition, we built manually HMM profiles for each of the GH16, thanks to the public CAZy database and their recent work on GH16 (Viborg et al., 2019). Thus, we were able to assign the enzymes suspected of being responsible for polysaccharide branching in Stramenopila to the GH16 subfamily 2. In addition, we checked a high number of putative beta-glucan candidate metabolic enzymes proposed in the literature for beta-glucan polysaccharide storage metabolism (Michel et al., 2010; O’Neill et al., 2015; Kuhaudomlarp et al., 2019) and tried to correlate them with the presence of the GT48 and GH16 subfamily 2 (Table 1 and Supplementary Table S1). Astonishingly, we did not find a correlation (absence in several Gyrista) with the GH16 subfamily 3 and 4 sequences despite these having been reported to encode laminarinase activities (Viborg et al., 2019). We specifically found instead two different candidate enzymes correlating with the distribution of the GT48 in Stramenopila: GH81 and GH5_33. These had both been previously reported as possibly involved in laminarin hydrolysis despite the absence of clear endomembrane targeting sequences (Michel et al., 2010; O’Neill et al., 2015). In addition, in agreement with recent proposals (Kuhaudomlarp et al., 2019), we consider that the GH161 phosphorylase, despite its absence from oomycetes genomes, remains possibly involved in phosphorolysis of beta-glucans albeit selectively in Ochrophytes, mirroring the role of the GT35 glycogen/starch phosphorylase in these beta-glucan accumulating lineages. Nevertheless, if we assume this function for ochrophyte polysaccharide breakdown, we must conclude that at variance with what has been demonstrated for alpha-glucan metabolism, phosphorolysis remains dispensable for beta-glucan metabolism as exemplified by oomycetes since no other candidate beta-glucan phosphorylases from the family GH94 and GH149 has been revealed in Stramenopila.

In addition, we have analyzed the subcellular localization and presence of transmembrane domains, on the subset of activities suggested in our study to be involved in beta-glucan metabolism (GH81 and GH5_33), and compared the analysis to results obtained with activities previously established as involved (GT48, GH16_2, and GH161) (Supplementary Table S2). We observed that prediction of the secretion signal, expected to deliver proteins to vacuoles (Gruber and Kroth, 2017), was not conserved among the different sequences and was not informative enough to help rule out their vacuolar localization. For instance, GT48 sequences were mainly lacking secretion signals. However, analysis of transmembrane domains shows their clear presence in GT48 enzymes, which strongly suggest their presence in the tonoplast membrane. For all other activities (GH81, GH5_33, GH16_2, and GH161), because only a few transmembrane domains were detected and that most of the sequences did not display a clear targeting signal, those results prevent us from drawing clear conclusions.

### Alpha-Glucan Metabolism in Stramenopila Is From an Ancient Eukaryotic Origin

**FIGURE 2:**
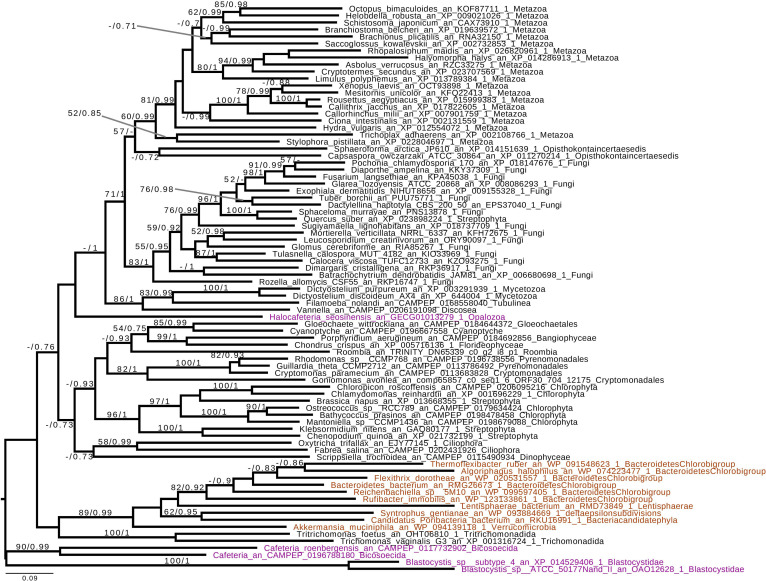
Phylogenetic analysis of branching enzyme, Glycoside Hydrolase family 13 subfamily 8 (GH13_8). The tree displayed is midpoint rooted and represents the consensus tree obtained with Phylobayes 4.1 with ML bootstrap values drawn from 100 bootstraps repetition with IQTREE (left) and Bayesian posterior probabilities (right) mapped onto the nodes. Bootstrap values >50% are shown, while only posterior probabilities >0.6 are shown. The scale bar shows the inferred number of amino acid substitutions per site. Sequences are highlighted in purple for Stramenopila, brown for Bacteria, while everything else is in black. Sequence names are composed of the organism name, the accession number, and their clades. We can see that Blastocystis and Cafeteria group together, however, with a low bootstrap and posterior probabilities. In addition, Halocafeteria was not found inside a monophyletic group, but was found among other eukaryotes.

**FIGURE 3:**
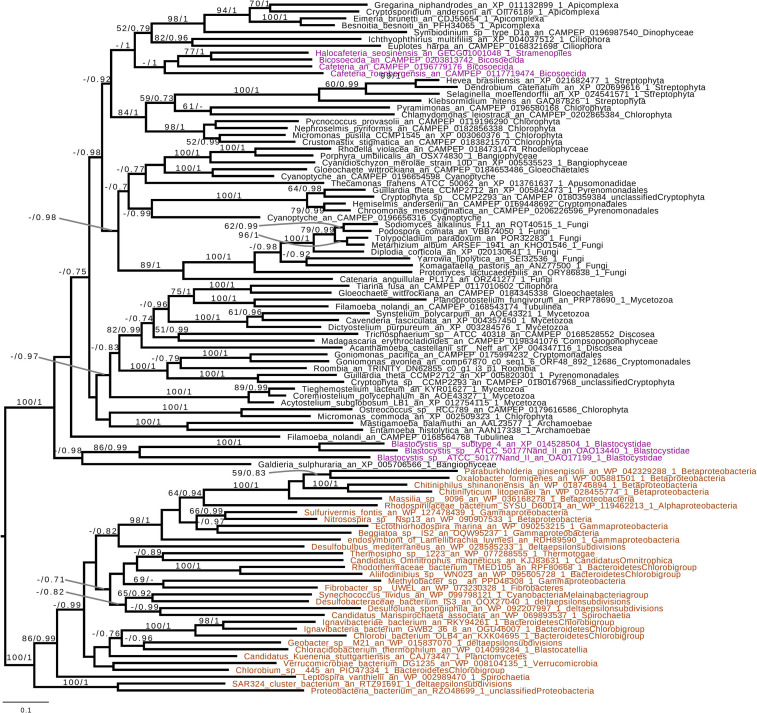
Phylogenetic analysis of glycogen phosphorylase (GT35 family). The tree displayed is midpoint rooted and represents the consensus tree obtained with Phylobayes 4.1 with ML bootstrap values drawn from 100 bootstraps repetition with IQTREE (left) and Bayesian posterior probabilities (right) mapped onto the nodes. Bootstrap values >50% are shown, while only posterior probabilities >0.6 are shown. The scale bar shows the inferred number of amino acid substitutions per site. Sequences are highlighted in purple for Stramenopila, brown for Bacteria, while everything else is in black. Sequence names are composed of the organism name, the accession number, and their clades. We can see that Bicosoecida, Halocafeteria, and Cafeteria group together, with a high posterior probability (pp = 1). However, Blastocystis was not found inside a monophyletic group with Stramenopila but was found among other eukaryotes. The Blastocystis position is probably due to the fast-evolving sequence in this organism as it has been observed in several studies (Eme et al., 2017; Moreira and López-García, 2017).

Based on the presence of alpha-glucan metabolism in both Opalozoa genomes analyzed, we tried to understand if enzymes were either acquired in a common ancestor or separately in those organisms. We, thus, performed a phylogenetic analysis of alpha-glucan storage metabolism enzymes. Using the MMETSP database (Keeling et al., 2014), we were able to find some enzyme sequences from the Cafeteria and Bicosoecida genera, to strengthen our analysis. We observed two patterns: on the one hand, for two enzymes, indirect debranching enzyme (GH13_25-GH133) (Supplementary Figure S1) and branching enzyme (GH13_8) (Figure 2), we were able to find Blastocystis and Halocafeteria or Cafeteria grouping together. However, in every case, bootstraps (BS) and posterior probabilities (pp) were low. In addition, in these two trees, at least one sequence of Stramenopila was not found inside the corresponding monophyletic group. On the other hand, in the case of both GT35 (Figure 3) and GT5 (Supplementary Figure S2), Blastocystis was not convincingly grouping with other Opalozoa but remained closely associated with eukaryotes although not with the metazoan enzymes. Although this fails to strengthen the case for a monophyletic origin of glycogen metabolism in Bigyra, it nevertheless does not reject it and further precludes possible LGTs from the animal hosts or from gut bacteria to the intestinal protist Blastocystis. Taken together, these results remain in agreement with a single ancient origin of alpha-glucan metabolism in Stramenopila.

### Beta-Glucan Metabolism Displays a Vertical Inheritance in Gyrista

**FIGURE 4:**
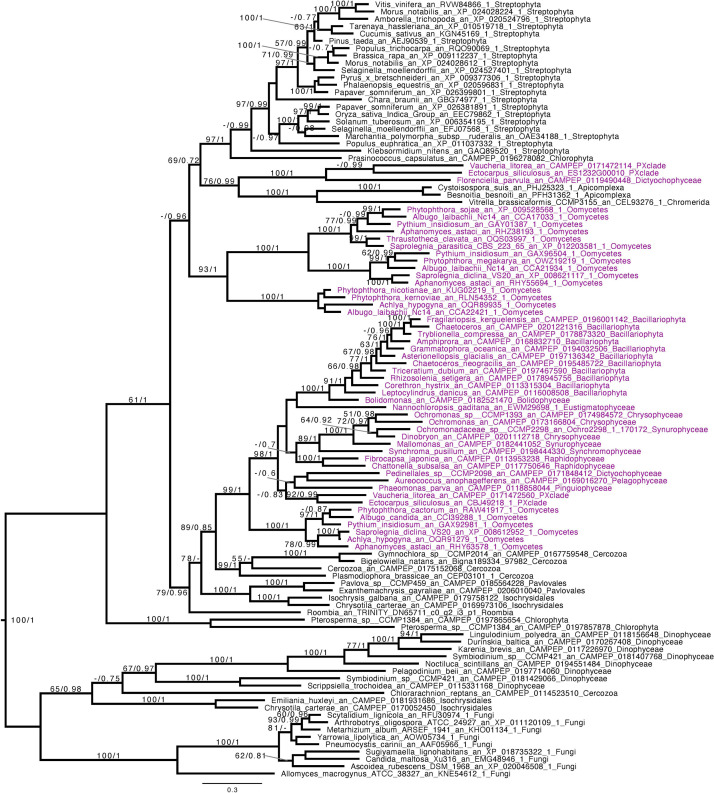
Phylogenetic analysis of GT48. The tree displayed is midpoint rooted and represents the consensus tree obtained with Phylobayes 4.1 with ML bootstrap values drawn from 100 bootstraps repetition with IQTREE (left) and Bayesian posterior probabilities (right) mapped onto the nodes. Bootstrap values >50% are shown, while only posterior probabilities >0.6 are shown. The scale bar shows the inferred number of amino acid substitutions per site. Sequences are highlighted in purple for Stramenopila, while everything else is in black. Sequence names are composed of the organism name, the accession number, and their clades. We can observe a strongly supported group with all Gyrista (BS = 99, pp = 1), with inside the expected topology based on Stramenopila phylogeny. In addition, among those sequences we find the characterized enzyme from *P. tricornutum*. Then, this group of sequence is likely to be the one involved in laminarin biosynthesis. Moreover, we can find close to this Gyrista group several sequences from both Haptophyta and Cercozoa (BS = 79, pp = 0.96), probably also involved in storage polysaccharide metabolism.

**FIGURE 5:**
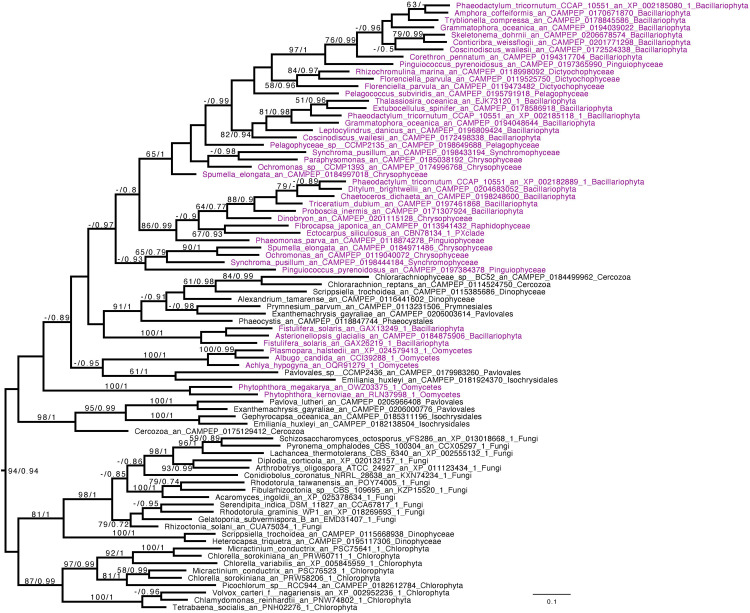
Phylogenetic analysis of GH16_2. The tree displayed is manually rooted according to the topology obtained in Supplementary Figure S3. It represents the consensus tree obtained with Phylobayes 4.1 with ML bootstrap values drawn from 100 bootstraps repetition with IQTREE (left) and Bayesian posterior probabilities (right) mapped onto the nodes. Bootstrap values (BS) >50 are shown, while only posterior probabilities (pp) >0.6 are shown. The scale bar shows the inferred number of amino acid substitutions per site. Sequences are highlighted in purple for Stramenopila, while everything else is in black. Sequences names are composed of the organism name, the accession number, and their clades. GH16_2 from the Stramenopila group together with sequences from Haptista and Cercozoa with a BS = 94 and a pp = 0.94, mirroring the topology from the GT48 phylogenetic tree.

We performed phylogenetic analysis for the two enzymes that have been proven or are very highly suspected to be active in chrysolaminarin metabolism. We reveal that both the GT48 (Figure 4) and the GH16 subfamily 2 (Figure 5 and Supplementary Figure S3) display a close relationship between Gyrista, Haptophyta, and Cercozoa. Indeed, in the GH16 subfamily 2 phylogeny, Haptophyta displays sisterhood and is even embedded inside the Stramenopila group as they are grouping with BS = 94 and pp = 0.94. In addition, the GT48 phylogeny possessed a somehow similar topology, with slight differences, the Cercozoa being the sister group (BS = 89, pp = 0.85) while the Haptophyta grouped with them with a BS of 78. Finally, we can observe in both phylogenies two groups consisting of Fungi and Viridiplantae (Chlorophyta or Streptophyta, respectively, in GH16_2 and GT48 trees). In both cases, the enzymes are demonstrated to be used in cell wall metabolism rather than storage as beta-glucan storage polysaccharides (Bowen and Wheals, 2004; Nishikawa et al., 2005).

Nevertheless, they should have the same type of enzyme catalytic activity as in Stramenopila. It must be stressed that some Stramenopila also synthesize beta-glucans in their cell wall and that the presence of different Stramenopila subgroups in the phylogeny may be understood in this light. The GH81 phylogenetic tree (Supplementary Figure S4), and particularly part II, also displays the relation between Stramenopila, Cercozoa, and Haptophyta, but with a lower robustness (BS = 51 and pp = 0.88). However, the GH161 putative beta-glucan phosphorylase phylogenetic tree (Supplementary Figure S5) displays a totally different topology with Stramenopila and Dinoflagellate having exchanged their gene probably multiple times, while the sequence seems to originate from Bacteria. Finally, the GH5_33 (Supplementary Figure S6) enzyme displays a topology analogous to those of the GH16_2 and GT48 enzymes (with organisms from Haptophyta and Cercozoa), albeit with some additional complexities including the additional presence of Ochromonas, Ochromonadaceae, and ciliates on the top of the tree and the absence of plant sequences.

## Discussion

### Putative New Enzymes of Beta-Glucan Metabolism

Beta-glucan storage polysaccharide metabolism remains an understudied pathway. Indeed, in Stramenopila, only two enzymes are known to be clearly involved in Chrysolaminarin biosynthesis, GT48 and GH16_2, and among these two, only GT48 was functionally fully demonstrated to be involved. By looking at the sequence distributions in Stramenopila using the CAZy database as basic knowledge to determine candidates involved in beta-glucan metabolism and the freely available HMM profile from the automated Carbohydrate-active enzyme annotation (dbCAN) to further assign the sequences detected to their corresponding enzyme subfamilies, we were able to propose the possible involvement of both the GH81 and GH5_33 enzymes. Interestingly, the GH81 phylogeny when concentrated around the Stramenopila group II (Supplementary Figure S4) displays a similar phylogenetic pattern to those seen with both GT48 and GH16_2, with the presence of Haptophyta and Cercozoa. Moreover, the GH5_33 phylogeny is also somewhat comparable to the topology of GT48 and GH16_2, thus for each enzyme (GH5_33 and GH81), the two correlations: on the one hand, co-occurrence of the enzymes (Table 1), and, on the other hand, the topology of their phylogeny suggests that they have evolved simultaneously and could be involved in chrysolaminarin catabolism.

In addition, phosphorolysis of storage polysaccharides was recently inferred to play a role in beta-glucan catabolism by generating glucose-1-phosphate, thereby retaining some of the free energy required for synthesis. We report here the distribution in Stramenopila of the new GH161 family (Kuhaudomlarp et al., 2019; Table 1), which could be involved in Chrysolaminarin catabolism. Surprisingly, this enzyme seems to be only present in Ochrophyta. In diatoms, it was proposed by Kroth et al. (2008) that absence of phosphorolysis was compensated by the presence of glucokinase; however, because of the presence of GH161, this could very well not be entirely true. However, the absence of GH161 in oomycetes suggests that the presence of such an enzyme could be dispensable and glucokinase, for instance, could be sufficient to generate the required sugar phosphate. This result also suggests that GH161 may have been acquired in an endosymbiotic context and is probably favored by the presence of glycolysis in two different compartments (i.e., cytosol and plastid). Moreover, GH161 sequences from the Ochrophyta group with bacterial sequences, which suggest a lateral gene transfer from Bacteria to an Ochrophyta ancestor. Finally, even if we did not find a sequence in *E. siliculosus* (Supplementary Figure S5), the presence of GH161 enzymes in the brown alga sister group Raphidophyceae (Fibrocapsa) suggests that the gene was either simply not found or lost in *Ectocarpus*, further suggesting the dispensable nature of phosphorolysis in beta-glucan metabolism.

### Possible Relative Merits of Alpha- vs. Beta-Glucan Water-Soluble Polysaccharides

While soluble alpha-glucan storage polysaccharides are always synthesized in the cytosol and degraded in both the cytosol and lysosome, the soluble beta-glucan storage polysaccharides such as Chrysolaminarin are synthesized, kept, and degraded in dedicated vacuoles. A rationale behind this observation may be found in the structure of those polysaccharides; indeed, laminarin displays a molecular weight varying from 3 to 10 kDa (Kadam et al., 2015) while that of glycogen is usually between 10^3^ and 10^4^ kDa (Sullivan et al., 2012), involving very important differences (up to three orders of magnitude) in their respective degree of glucose polymerization, which directly impacts the osmotic pressure of the cellular compartment where they are synthesized. Thereby, the high degree of polymerization of glycogen makes it way more osmotically tolerable for the fragile cytosol of eukaryotes while Chrysolaminarin-type polysaccharide/oligosaccharides can represent, if in high amounts, a problem that is, at least, partly resolved by its vacuolar localization. The penalties and benefits ensuing from storage compounds of, respectively, high and low degree of polymerization will thus vary greatly with the presence or absence of a rigid vegetative cell wall and with the subcellular localization of such compounds. Despite these considerations, it remains, however, very hard to understand what has apparently favored the selection and replacement of alpha-glucan metabolism by that of beta-glucans in a majority of rhizarians and most stramenopiles over the maintenance of glycogen metabolism in all alveolates and opalozoans.

### Origin of Metabolism in Stramenopila

If we remove storage lipid metabolism from our reasoning and restrict our comparisons to alpha- and beta-glucan metabolism as carbon storage material, we observe that those pathways seem mutually exclusive in Stramenopila as they are in all eukaryotes. While beta-glucan is found in Gyrista, alpha-glucan is found in Opalozoan which begs the question of the ancestrality of both types of metabolism. To shed light on such issues, we focused our attention to our phylogenetic analysis. This shows that in the alpha-glucan metabolism iDBE (GH13_25-GH133) from Stramenopila (Supplementary Figure S1) seems to form a cluster with other Alveolata, the closest related group from Stramenopila (with low statistical value pp = 0.7, BS < 50). The same observation can be made for the GT35 phylogeny of Halocafeteria and Cafeteria (Figure 3) (pp = 1), also suggesting a vertical inheritance. We should note that for GT35, Blastocystis is clustering more distantly, which could be due to the fast-evolving sequence in this organism (Eme et al., 2017; Moreira and López-García, 2017). In addition, both GH13_8 (Figure 2) and GT5 (Supplementary Figure S2) are found close to other eukaryotic sequences. Altogether, these phylogenies suggest that the Stramenopila ancestors initially had this ancient eukaryotic metabolism.

Beta-glucan metabolism shows a common pattern between GT48 (Figure 4), GH16_2 (Figure 5), and GH81 part II (Supplementary Figure S4), with a clustering with Haptophyta and Cercozoa. In addition, the GH5_33 phylogenies display a related topology (Supplementary Figure S6), while the group on the top is composed of both Ochromonas and Ochromonadaceae with ciliates that are associated with a long branch that could indicate another function in this particular cluster. On the contrary, GH161 (Supplementary Figure S5) seems to have been acquired specifically in the Ochrophyta ancestor by lateral gene transfer from Bacteria.

These results on beta-glucan storage metabolism in Stramenopila might also suggest an ancient relation between Stramenopila and beta-glucan polysaccharide. In particular, since Rhizaria and Haptista are closely related groups to Stramenopila in eukaryote phylogeny (Burki et al., 2016). A single evolution of storage beta-glucan metabolism at the base of the SAR group followed by segregation of alpha- and beta-glucan metabolisms leading to maintenance of glycogen and loss of beta-glucans in alveolate and opalozoa can at least be proposed.

Alternatively, the fact that, among Stramenopiles, only Gyrista can store beta-glucan might indicate that this metabolism could have been gained through the endosymbiosis process of Haptophyta as it has been proposed in the serial hypothesis (Baurain et al., 2010). In this case, however, we would have to infer that Oomycetes originated after the secondary endosymbiosis event that would have generated the Gyrista.

Altogether, these results point toward an acquisition of storage beta-glucan polysaccharide metabolism through gene inheritance rather than from a specific metabolic sub-functionalization that occurred specifically in Stramenopila. This result suggests that re-targeting of cell wall metabolism, as proposed in Kroth et al. (2008), to storage polysaccharide metabolism did not occur in Stramenopila, but could have nevertheless occurred in other organisms.

### Ancient Evolution of Beta and Alpha Storage Polysaccharide Metabolisms

Understanding storage polysaccharide metabolism distribution in Stramenopiles requires a better understanding of their ancestral status. To address this question, we investigated the metabolic distribution of alpha- and beta-glucans in eukaryotes. Interestingly, alpha-glucan storage polysaccharides seem to be found in all eukaryotes: unikonts (Amoebozoan, Obazoa) and bikonts (SAR, Archaeplastida, Cryptista, Haptista, Excavata) alike (Ball et al., 2011). In particular, this work shows a very ancient relation between Stramenopila and alpha-glucan storage metabolism, while soluble beta-glucan storage polysaccharides seem to be only found in some bikonts and never found in unikonts.

In addition, although cell wall beta-1,3 glucan polysaccharides are widespread in bikonts and unikonts, the beta-1,3-glucan storage polysaccharides are limited to bikonts, suggesting a scenario whereby beta-glucan storage metabolism may have evolved from preexisting cell wall polysaccharide metabolism at the base of the SAR and haptophytes while alpha-glucan storage metabolism evolved during eukaryogenesis and thus pre-existed in both the ancestor of the SAR and that of the haptophytes. Hence, ancestors of such organisms could have possessed both alpha- and beta-glucan storage metabolisms. Indeed, this exceptional situation seems to exist in both Goniomonas (Cenci et al., 2018b) and Emiliana (data not published).

**FIGURE 6:**
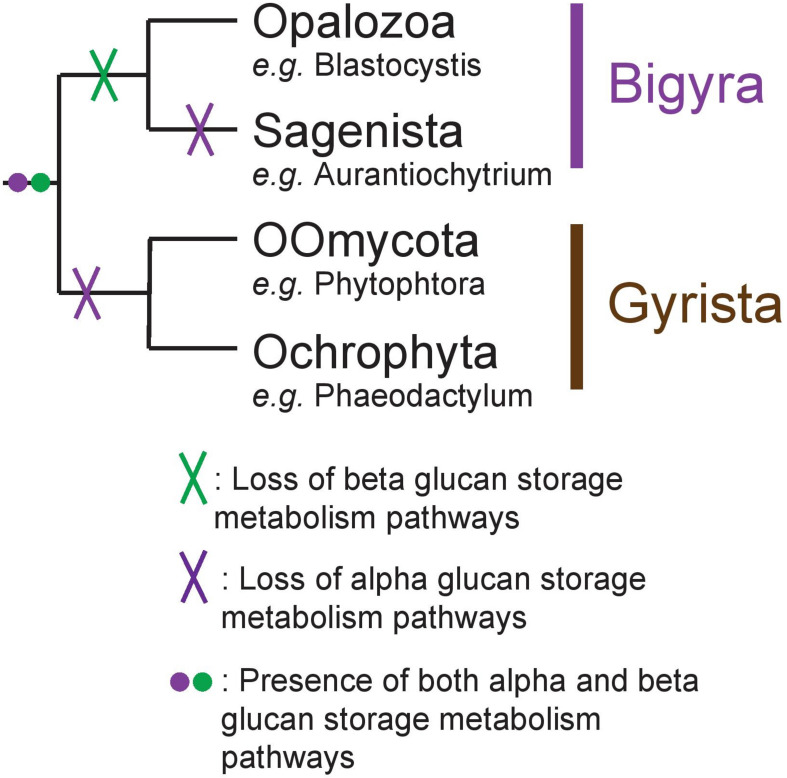
Schematic representation of the Stramenopila evolution tree with the hypothesis defended here with the presence of both alpha- and beta-glucan storage polysaccharide metabolisms in the ancestor, represented by the purple and green dot, respectively. Their respective loss is indicated by a cross on the branch: in Bigyra, the loss of beta-glucan metabolism is indicated by a green cross, while alpha-glucan metabolism has been lost twice in Gyrista lineages as well as in Sagenista (purple cross).

While we acknowledge the fact that scarcity of genomes in Opalozoa could induce biases in our interpretation, we propose nevertheless that, in Stramenopila, there was an ancient presence of both alpha- and beta-glucan storage polysaccharide metabolism (Figure 6). This was followed by loss of beta-glucan storage metabolism in some lineages and of loss alpha-glucan metabolism in others while a few storage lipid accumulating organisms lost both.

## Data Availability Statement

The original contributions presented in the study are included in the article/Supplementary Material, further inquiries can be directed to the corresponding author/s. Publicly available datasets were analyzed in this study. Phylogenetic datasets analyzed are available at https://doi.org/10.5061/dryad.dv41ns1x3.

## Author Contributions

UC and SB designed the project. MCh and UC performed the annotation. MCh, ML, LF, and UC performed the phylogenetic analysis. MCh, ML, LF, MCo, CC, SB, and UC analyzed the results. UC and SB wrote the manuscript. All authors read and accepted the final manuscript.

## Conflict of Interest

The authors declare that the research was conducted in the absence of any commercial or financial relationships that could be construed as a potential conflict of interest.

## Supplementary Material

Supplementary Figure 1 Phylogenetic analysis of GH13_25 (iDBE).

Supplementary Figure 2 Phylogenetic analysis of GT5.

Supplementary Figure 3 Phylogenetic analysis of GH16_2 rooted with other GH16 sequences.

Supplementary Figure 4 Phylogenetic analysis of GH81.

Supplementary Figure 5 Phylogenetic analysis of GH161.

Supplementary Figure 6 Phylogenetic analysis of GH5_33.

Supplementary Table 1 Table of CAZy families and subfamilies found in 17 Stramenopila genomes, with the accession number for each of the annotated sequences.

Supplementary Table 2 Table of targeting prediction and transmembrane domains for GT48, GH16_2, GH81, GH5_33, and GH161.
