# Supplementary material for: Retracing Storage Polysaccharide Evolution in Stramenopila
Source: Front Plant Sci. 2021 Mar 3;12:629045. doi: 10.3389/fpls.2021.629045 (PMC7965971; doi:10.3389/fpls.2021.629045)
Supplement: Supplementary Figure 1 — Phylogenetic analysis of GH13_25 (iDBE). [file Data_Sheet_1.DOCX]

**Supplementary data**

Figure 1S: Phylogenetic analysis of GH13_25-GH133 proteins (iDBE). The tree displayed is midpoint rooted and represents the consensus tree obtained with Phylobayes 4.1 with ML bootstrap values drawn from 100 bootstraps repetition with IQTREE (left) and Bayesian posterior probabilities (right) mapped onto the nodes. Bootstrap values (BS) >50 are shown, while only posterior probabilities (pp) >0.6 are shown. The scale bar shows the inferred number of amino acid substitutions per site. Sequences are highlighted in purple for Stramenopila, while everything else is in black. Sequences names are composed of the organism name, the accession number and their clades. All Bigyra are grouping together with a low pp = 0.7, and as known for the iDBE only other Eukaryota are found in the tree, indicating deep origin putatively tracing back the origin of the gene to the last eukaryotic common ancestor.

Figure 2S: Phylogenetic analysis of GT5. The tree displayed is midpoint rooted and represents the consensus tree obtained with Phylobayes 4.1 with ML bootstrap values drawn from 100 bootstraps repetition with IQTREE (left) and Bayesian posterior probabilities (right) mapped onto the nodes. Bootstrap values (BS) >50 are shown, while only posterior probabilities (pp) >0.6 are shown. The scale bar shows the inferred number of amino acid substitutions per site. Sequences are highlighted in purple for Stramenopila, brown for Bacteria, while everything else is in black. Sequences names are composed of the organism name, the accession number and their clades. Sequences from Bigyra are found embedded within eukaryotes sequences (BS = 100, pp = 1). This suggests a most likely vertical inheritance from a eukaryotic ancestor.

Figure 3S: Phylogenetic analysis of GH16_2 rooted with other GH16 sequences. The tree displayed is midpoint rooted and was obtained using Maximum Likelihood approach with IQTREE the values indicated on each branch is drawn from 1000 ultrafast bootstraps repetition. The scale bar shows the inferred number of amino acid substitutions per site. Sequences are highlighted in purple for Stramenopila, while everything else is in black. Sequences names are composed of the organism name, the accession number and their clades. Thanks to the rooting, we can see that the tree is oriented as in Figure 6 and we confirm our observation.

Figure 4S: Phylogenetic analysis of GH81. The tree displayed is midpoint rooted and represents the consensus tree obtained with Phylobayes 4.1 with ML bootstrap values drawn from 100 bootstraps repetition with IQTREE (left) and Bayesian posterior probabilities (right) mapped onto the nodes. Bootstrap values (BS) >50 are shown, while only posterior probabilities (pp) >0.6 are shown. The scale bar shows the inferred number of amino acid substitutions per site. Sequences are highlighted in purple for Stramenopila, brown for Bacteria, while everything else is in black. Sequences names are composed of the organism name, the accession number and their clades. The phylogenetic tree of GH81 and especially the part II displays the relation between Stramenopila, Cercozoa and Haptophyta, however, with a lower robustness (BS = 51 and pp = 0.88).

Figure 5S: Phylogenetic analysis of GH161. The tree displayed is midpoint rooted and represents the consensus tree obtained with Phylobayes 4.1 with ML bootstrap values drawn from 100 bootstraps repetition with IQTREE (left) and Bayesian posterior probabilities (right) mapped onto the nodes. Bootstrap values (BS) >50 are shown, while only posterior probabilities (pp) >0.6 are shown. The scale bar shows the inferred number of amino acid substitutions per site. Sequences are highlighted in purple for Stramenopila, while everything else is in black. Sequences names are composed of the organism name, the accession number and their clades. GH161 sequences from Stramenopila possesses a totally different topology compared to GH16_2 or GT48, with Stramenopila and Dinoflagellata having exchanged their gene probably multiple times, while the sequence seems to originate from Bacteria, indicating a late acquisition of this sequence compared to other beta glucan storage polysaccharide metabolism enzymes.

Figure 6S: Phylogenetic analysis of GH5_33. The tree displayed is midpoint rooted and represents the consensus tree obtained with Phylobayes 4.1 with ML bootstrap values drawn from 100 bootstraps repetition with IQTREE (left) and Bayesian posterior probabilities (right) mapped onto the nodes. Bootstrap values (BS) >50 are shown, while only posterior probabilities (pp) >0.6 are shown. The scale bar shows the inferred number of amino acid substitutions per site. Sequences are highlighted in purple for Stramenopila, while everything else is in black. Sequences names are composed of the organism name, the accession number and their clades. This tree has a slightly different topology, indeed if we can find almost the same topology as GH16_2 and GT48, there is on the top of the tree a group with Ochromonas, Ochromonadaceae and ciliates preventing from easily comparing topology with other beta glucan storage polysaccharide metabolism enzymes and suggest a more complicated story for this last part of the tree.
